# Ensemble agent based machine learning approach for energy efficient attack detection and prevention in MANETs

**DOI:** 10.1038/s41598-025-31628-4

**Published:** 2025-12-18

**Authors:** Pratiksha Nigam, Ajay Tiwari, Tarun Dhar Diwan, Mahdal Miroslav, SP Samal

**Affiliations:** 1 School of computer science and IT, Devi Ahilya Vishwavidyalaya, Indore, India; 2 Department of Computer science and Engineering, Atal Bihari Vajpayee University, Bilaspur, India; 3 Faculty of Mechanical Engineering, VSB-Technical University of Ostrava, Ostrava, Poruba, 70800 Czech Republic; 4 Department of Biosciences, Saveetha School of Engineering, Saveetha Institute of Medical and Technical Sciences, Chennai, 602105 India

**Keywords:** MANETs, DoS attacks, Detection, ML, Blackhole, Gray hole, Engineering, Theory and computation

## Abstract

Mobile Ad hoc Networks (MANETs) represent a decentralized and self-tuning network paradigm that relies on routing protocols to transmit data from source to destination. However, the absence of a fixed infrastructure makes MANETs vulnerable to various security threats, including blackhole and gray hole attacks. Addressing these vulnerabilities is critical to ensuring the reliability and security of MANETs. The paper proposes an agent-based approach for effectively identifying and preventing such attacks within the MANET environment. Unlike existing static or centralized models, agent-based approach deploys dedicated agent nodes in each cluster for real-time monitoring and classification of malicious behaviour. Furthermore, the paper introduces an energy-efficient optimum clustering method, leveraging ensemble clustering-based optimization techniques, to select cluster heads responsible for data aggregation. The combination of optimal clustering and agent-based attack detection enhances the overall security and performance of the MANET. This also significantly improving energy efficiency and data aggregation reliability. Each cluster in the proposed model is equipped with an attack detection agent node, which plays a critical role in identifying suspicious, blackhole, wormhole, and normal nodes within the incoming traffic. This proactive detection mechanism ensures timely response and mitigation of potential security threats. The development of an ensemble-based clustering optimization technique to enhance energy efficiency and improve data aggregation. In addition to the detection mechanism, the study performs a comprehensive comparison of multiple machine learning algorithms. This comparison aims to determine the most effective models for accurate attack identification and trust score generation for network nodes. This determines the most effective algorithms based on accuracy and computational cost by enabling more accurate threat identification and trust-based routing decision. By combining agent-based attack detection, energy-efficient clustering, and intelligent machine learning models, this research work offers a comprehensive and robust solution to enhance the security and reliability of MANETs. Experimental results on simulated MANET environments demonstrate that the proposed approach significantly improves detection accuracy and enhances network lifetime and throughput compared to existing methods. Specifically, proposed approach achieved a throughput of 93 Kbps. This shows approx. 8% improvement over existing approach. The results demonstrate the effectiveness of the proposed approach in providing valuable insights for future research in securing MANETs.

## Introduction

Mobile ad hoc network (MANET) is a network-type in which there is no centralized network and all nodes are self-tuned^1^. But each node is constrained with limited range for data transmission from source to destination. Therefore, routing protocols are used to transmit data from source to destination. But while routing entire network is considered to be as normal which is practically impossible. As MANET is being used in several applications such as disaster management^2^, Military^3^, medical and research^4^, smart city^5^, etc. For data transmission routing protocol is divided into three mechanisms i.e., proactive^6^, reactive^7^ and hybrid^8^. In dynamic network situation, the nodes are mobile in nature, that’s why it is vulnerable to network attacks. Some of the commonly occurring attack in dynamic MANET are gray hole^9^, black hole^10^, and selective packet drop attack^11^. These attacks cause packet drop during data communication. Hence, it is quite challenging task to identify and classify these attacks in MANET. Many researchers have proposed various solutions to detect and prevent of these attacks. Most of the research contributions are dedicated to handle single attack condition, i.e., either blackhole or gray hole. There are very few contributions presented that can identify multiple attacks simultaneously. It is quite difficult to identify these attacks at route discovery time. Therefore, this work is dedicated to detect and prevent packet drop attacks including gray hole and black hole attack in MANET taking the usage of machine learning approaches. For this, Table 1 presents the investigated research questions along with its respective research contributes.

**Table 1:** Research questions and respective Contributions.

Research questions	Research contributions
How machine learning has emerged in the DoS attack detection?	To prepare the evolution and systematic meta-analysis of blackhole and Gray hole attack detection techniques.
How DoS attacks in MANET be identified?	For identification and categorization of DoS attack machine learning based detection technique is used.
How network’s energy efficiency is considered?	For designing of energy efficient MANET architecture, a multi-objective ensemble clustering-based optimal cluster formation and selection strategy is deployed.

The rest part of the paper has been organized as: Sect. “Related work” presents the related study for black hole detection and Gray hole attack detection. For systematic meta-analysis, optimization and machine learning approaches are discussed. Section “Black hole attack detection techniques” presents the materials and methods for detection and prevention of these attacks. This section also presented the comparative state-of-art. Section “Gray hole attack detection techniques” presents results and discussions. The conclusion and future scope of the paper are presented in Sect. “DoS attacks in MANET and its detection” at last.

## Related work

### Black hole attack detection techniques

The routing-disrupting attack also known as a black hole attack has the potential to cause significant harm to the network. Therefore, it is necessary to have a reliable technique for identifying black hole attacks. Using a linear regression model, Pullagura et al.^12^ calculated the maximum possible destination sequence. The performance of normal, black hole-based, and black hole-detected routing techniques are all compared with one another. The results of the simulations demonstrate that the implemented strategy improves network performance by boosting QoS. By using “cross-feature analysis,” a new data mining technique described by Huang et al.^13^ can record the inter-feature association patterns seen in everyday traffic. These patterns can serve as reference points for normal profiles against which attacks can be identified. A new data mining technique called “cross-feature analysis” was presented by Huang et al.^13^ as a means of identifying the inter-feature correlation patterns that are present in normal traffic. In future, we hope to provide more researchers with comprehensive work. A new method for the detection of black hole attacks in mobile ad hoc networks was developed by Houda et al.^15^, which combines an Adaptive Neuro-Fuzzy Inference System (ANFIS) with Particle Swarm Optimization (PSO). Pawar et al.^16^ set out to introduce a cutting-edge, deep-learning-based solution for spotting and stopping wormhole and black hole attacks in WSN. Included in this process are phases like node assignment, data collecting, detecting wormhole and black hole attacks, as well as preventing them through optimal path communication. Taylor SailFish Optimizer (TaylorSFO) is a new approach presented by Kumar et al.^17^ for predicting blackhole attacks in WSN. The proposed Taylor-SFO combines the Taylor Series with the SailFish Optimizer to train the Deep stacked autoencoder. (SFO). WSN base stations use the newly designed Taylor-SFO to perform routing and blackhole attack detection. The suggested model has two parts: the first deals with routing, while the second detects blackhole attacks at the base station. Black hole attacks and wormhole attacks were both taken into account by Daniel et al.^18^. As input, we use the total number of sensor nodes in the wireless network. The loss of data packets and delays in transmission are two major concerns with a wormhole and black hole attacks. Deep neural networks were utilized in the development of the fuzzy imperialist competitive algorithm (DNN-FICA), which was created to get over these limitations and successfully detect an attacker node in a wireless network. When new data is added, machine learning (ML) automatically analyzes it based on previously stored data that has been labeled and examined for patterns. The three main branches of machine learning are unsupervised learning, reinforcement learning^9^, and supervised learning. To protect MANETs from Flooding and Blackhole Attacks, Shafi et al.^19^presented ML-AODV, an AODV routing protocol based on machine learning and trust. To prevent a flood attack in which a large number of routing packets are sent to non-existent destinations, it is vital to pick intermediary nodes in the network that are trustworthy. A number of node-specific parameters, including Link Expiration Time (LET), Hop Count (HC), and Residual Energy (RE), are taken into account in this regard. Kaur et al^20^. zeroed in on the Blackhole security threat; security is crucial from both the source and the destination nodes’ perspectives during data transfer. Moreover, WSNs are challenging to administer or construct, while networks built with the aid of ML are really straightforward. Gray hole attacks, flooding attacks, and Blackhole attacks were the focus of Kurtkoti et al.‘s^21^ research into their detection and mitigation. In the first stage, we assess how well various ML algorithms can spot these attacks, and accordingly, the study offers a method for protecting against them. In order to collect the WSN-DS dataset, a hostile node carrying out Gray hole or black hole attacks is artificially induced.

### Gray hole attack detection techniques

A gray hole attack is a denial-of-service technique that builds on the principles of a black hole attack. A partial packet drop attack is one in which just some of the data packets exchanged between two parties are lost. In the study, gray-hole nodes are recognized as malicious nodes because they act similarly to other nodes throughout the path-finding process. Cai et al.^22^ showed that a cross-layer adaptive strategy can be used to identify Gray and black hole attacks in an ad hoc network. In this study, within the network layer, we suggested a path-based approach to overhear the action of the following hop. This method conserves the hardware resources of the detecting node by avoiding sending out unnecessary control packets. With the goal of defeating both the cooperative gray hole attack and the black hole attack, Vishnu et al.^23^ presented a trust management technique based on Dempster-Shafer (D-S) evidence. By putting an emphasis on the direct trust value between neighbors, the suggested method can identify a single black hole attack. When defending against gray-hole attacks, historical evidence is also considered. The denial-of-service attack known as selective forwarding or gray hole attack was the subject of Liu et al.‘s^24^research. Data loss could cause significant damage when an attack is launched at the gateways of a WMN, which is where data tends to aggregate. Most current ideas are ineffectual against collusive attackers because they rely on detecting lone attackers via channel overhearing. In wireless ad hoc network design, the gray wolf optimization (GWO) with trust setup data aggregation was presented by Vatambeti et al^25^.. Energy-efficient techniques were given by Dongare et al.^26^ to reduce the negative effects of both types of attacks on the cluster head selection mechanism. Here, we present an effective method for finding compromised nodes in WSNs and blocking them from participating in network communication. During the packet-sending phase, the trustworthy nodes are also chosen to take on the role of cluster leader. The Aware Fault Detection (IROA-LAFD) technique based on Improved Rider Optimization Algorithm network was presented by Janakiraman et al.^27^ to improve security by decreasing the impact of the gray hole and black hole attacks through increased network stability. This IROA-LAFD system aims to efficiently mitigate packet dropping by measures such as neighbor and attack detection, route discovery, secure packet transmission, link analysis, as well as IROA Algorithm-based link fault detection. In order to identify wormhole attacks in VANETs’ multi-hop communication, Singh et al.^28^ sought to employ the capabilities of machine learning. The author modeled the attack in a VANET by developing a multi-hop communication scenario based on the AODV routing protocol, which makes advantage of mobility traces. Shukla et al.^29^ proposed a deep learning-based approach using Mutation-Based Neural Networks and Artificial Bee Colony optimization to detect and prevent blackhole and grayhole attacks in MANETs. Their method improves node selection and outperforms existing techniques in attack mitigation.

## DoS attacks in MANET and its detection

Mobile Adhoc Network (MANET) is a kind of WSN that consist of set of mobile nodes connected together in infrastructure less type of network. Now-a-days, increasing usage of MANET made it one of the prevalent research topic among researchers because of its wide range of usage in critical applications including defence, medical, etc. With increasing usage, its security threat also increases^30–32^. In this paper, Denial of Service (DoS) attacks such as blackhole attacks (BHA) and Gray hole attacks (GHA) are considered. The main intension of these attack is to disrupt the flow within a network by blocking the services. Intruders or attackers prevent the sensor nodes to use their resources and decreases the network performance by creating packet loss, packet drop, delay, etc. Therefore, to ensure the security aspects, attack detection and prevention system need to be deployed. In this paper, machine learning approach has been applied to detect and prevent these attacks.

**Fig. 1:**
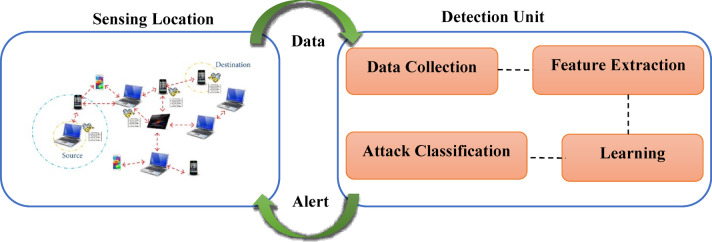
Attack Detection Scheme for MANET.

The general flow of machine learning based attack detection and prevention system is presented in Fig. 1. The system is divided into two main components: the “Sensing Location” and the “Detection Unit”. The sensing location represents the real-time MANET environment, consisting of multiple mobile nodes that communicate without fixed infrastructure. These nodes are vulnerable to Denial of Service (DoS) attacks like blackhole and gray hole attacks, where malicious nodes disrupt communication by dropping or misrouting packets. To counter these threats, the network continuously sends data to the detection unit, which consists of four main stages: data collection, feature extraction, learning, and attack classification. The detection unit collects network behaviour data, extracts relevant features, and uses machine learning models to learn and classify malicious behaviour. Once an attack is detected, an alert is sent back to the sensing location to take preventive actions such as isolating the malicious node or rerouting traffic.

However appropriate method is required to detect attacks among sensor nodes by using appropriate methods that considers the network restrictions and WSN traffic. Therefore, below we have discussed some of the machine learning approaches that are being implemented in this paper for deployment of attack detection unit.

## Materials and methods

**Fig. 2:**
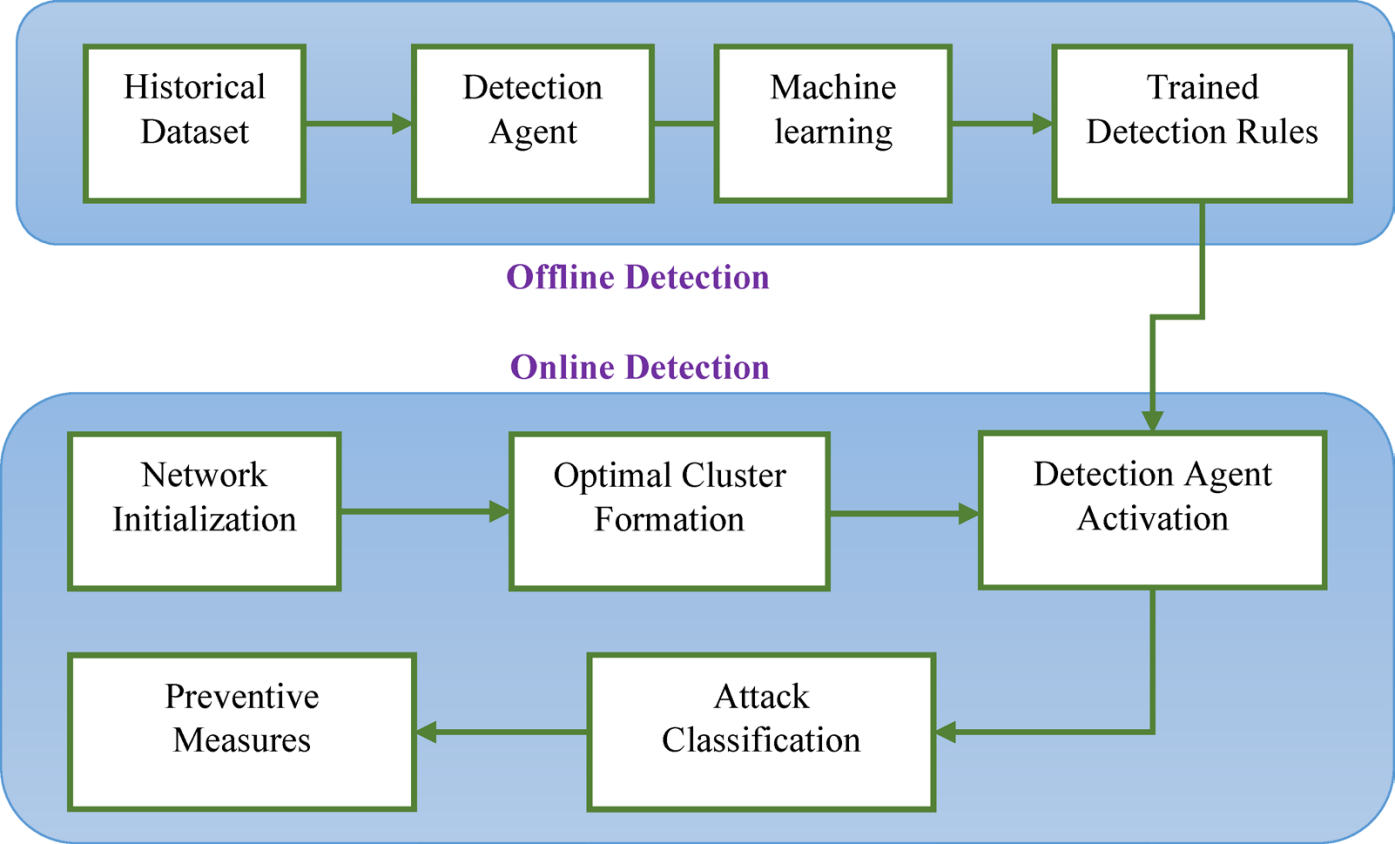
Proposed model.

**Fig. 3:**
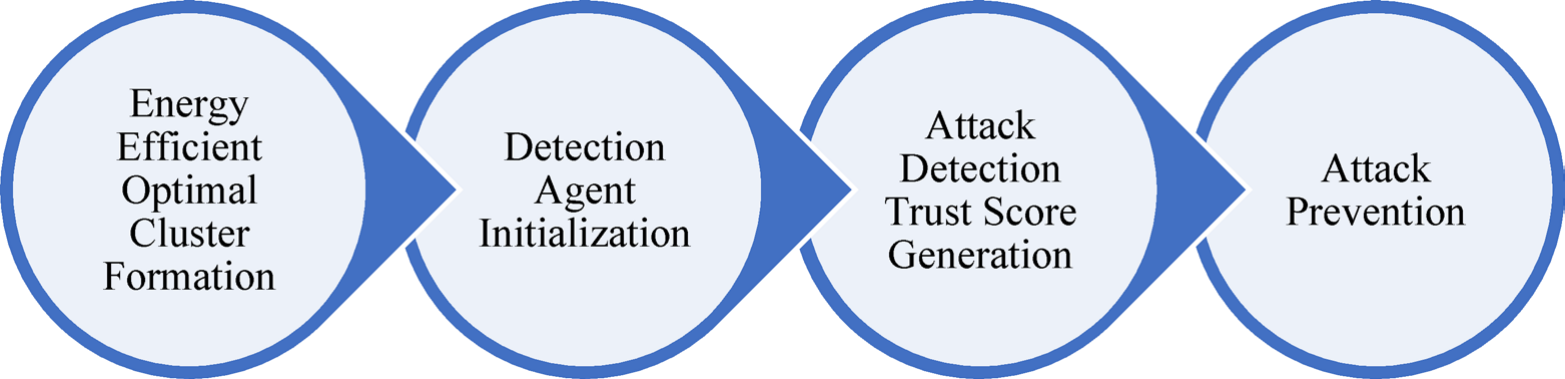
Working flow of proposed model.

This study proposes a novel approach for attack detection agent-based model is proposed to ensure security in MANET. The paper is designed to identify blackhole attacks (BHA) and Gray hole attacks (GHA) and provide preventive measures. The entire model is designed as an offline and online detection model which is presented in Fig. 2. In an offline model of detection, the paper implemented the machine learning model to train the detection agent that holds detection rules for the online mode of detection. In the proposed framework, both offline and online detection mechanisms are integrated to provide a comprehensive security solution for MANETs. The offline detection model functions as the training phase in which the historical dataset is used to train machine learning algorithms on known patterns of normal and malicious behaviour are learned. During this phase, key features are extracted, and classification models such as XGBoost are trained to develop accurate detection rules. These learned rules are then embedded within the detection agents for real-time application. Once the system transitions to the online detection mode, it operates in a live MANET environment where nodes dynamically form clusters through an ensemble-based clustering mechanism. When the network is initialized in online mode, the node with its configuration is sent to the optimal cluster formation unit that will optimally form clusters and select cluster heads. To enhance energy efficiency and scalability in MANETs, the proposed framework adopts a multi-objective ensemble clustering (MOEC) approach for optimal cluster formation and cluster head (CH) selection. The proposed method used ensemble of nature-inspired optimization algorithms by combining Particle Swarm Optimization (PSO) and Gray Wolf Optimization (GWO) to generate multiple clustering solutions. These are then fused into a consensus clustering to improve robustness and accuracy. CH selection is guided by three primary objective functions: (i) minimizing intra-cluster Euclidean distance to reduce communication overhead, (ii) minimizing mean adjacent distance relative to node radius to ensure compactness, and (iii) maximizing residual energy of the CH to prolong network lifetime. The fitness of each clustering solution is evaluated using these metrics, and the ensemble result ensures load balancing, energy efficiency, and improved network longevity. After the cluster head selection, one of the agent nodes is initialized as an attack detection agent node (DAN). DAN has the responsibility to detect malicious nodes that may cause BHA or GHA. DAN is integrated with machine learning-trained rules that predict the arrived node and its behaviours as attack type or normal. Upon detection of a malicious node, DAN not only raises an alert but also updates the node’s trust score. Nodes with consistently low trust scores are isolated from routing paths. This trust management system, integrated with ML-based detection enables both proactive prevention and reactive mitigation. Although Blackhole Attacks (BHA) and Gray hole Attacks (GHA) differ in behaviour. As the BHAs dropping all packets and GHAs dropping selectively, the proposed system uses a unified machine learning-based detection approach by focusing on key behavioural features that distinguish both attacks from normal node activity. During the offline training phase, the model is exposed to labelled examples of both BHA and GHA patterns. The XGBoost classifier is able to successfully differentiates between these different behavioural traits. Thus, this allows the unified model to accurately identify and classify both BHA and GHA attacks during runtime. This ensures the secure transmission of data. Therefore, the entire work is divided in four basic steps, as presented in Fig. 3. These steps are described as following:

### Energy efficient optimal cluster formation

In this stage, first of all, entire network is divided into clusters and optimal clusters are selected with cluster heads. In traditional clustering technique for WSN such as LEACH protocol^33^, there is random distribution of clusters which may lead to consumption of energy according to distance among CHs and base stations. Whereas in this paper, a nature-inspired optimization algorithm is presented for optimal cluster formation and head node selection. In this approach, the entire network is divided into number of clusters that is optimally selected by nature inspired optimization algorithm and each cluster contains its own cluster heads. Each cluster head (CH) has responsibility of data aggregation. Each sensor node forwards its sensed data to respective CH, which aggregates the sensed data from each node, residing in corresponding cluster, and forward to base station. The model-based presumption of the study was the following:

- In area $\:A$, $\:N$ number of sensor nodes are deployed. Location of these nodes are not fixed, they are located randomly.
- Each node comprised of unique ID. The base station is not energy constrained, whereas the nodes have a set the amount of energy.
- The network connections are assumed to be symmetric.
- Based on interaction distance, the transmission power is determined.

To evaluate the consumed energy by the sensor nodes while data communication is evaluated according to energy model. The energy cost is needed to be calculated during transmission and reception of data. Below Eq. (1) and Eq. (2) shows the mathematical expression for calculating energy during transmission of data bits and reception of data bits:

1 $$\:{E}_{tx}(m,s)=\:\left\{\begin{array}{c}{{d}_{l}E}_{selects}+\:{m\epsilon\:}_{fs}{{t}_{d}}^{2},\:\:\:\:\:\:\:\:\:\:\:\:\:\:\:\:\:\:\:{t}_{d}<{{t}_{d}}_{0}\\\:{{d}_{l}E}_{selects}+\:{m\epsilon\:}_{amp}{{t}_{d}}^{4}\:\:\:\:\:\:\:\:\:\:\:\:\:\:\:\:{t}_{d}\ge\:{{t}_{d}}_{0}\end{array}\right.$$

2 $$\:{E}_{rx}\left(m\right)=\:{d}_{l}{E}_{selects}$$

Here, message or data length is represented as $\:{d}_{l}$ and transmission range is evaluated as $\:{t}_{d}.\:$The energy required for transmission and reception of per unit length data is represented as E_selects_ whereas energy required by amplifier in free space as well as multiple path attenuation model is represented as $\:{\epsilon\:}_{fs}\:and\:{\epsilon\:}_{amp}$ respectively. But when the distance between receiver and transmitter is less than threshold value, $\:{{t}_{d}}_{0}$ then free space model is used and $\:{{t}_{d}}^{2}$ is evaluated as attenuation condition. While in multi-path attenuation condition $\:{{t}_{d}}^{4}$ is used. Therefore, for merging n-length data, energy required is represented as in Eq. (3):

3 $$\:{E}_{u}\left(m\right)=\:n{E}_{da}$$

Where E_da_ = The amount of power required for combining one unit of information.

The suggested methodology in this work selects cluster heads for clusters/groups established after the initiation step get finished. We have discussed the unequal clustering strategy^34^ since it has benefits such as enhanced network lifetime, load balancing, scalability, etc. In this research, we suggested a multi-objective ensemble clustering (EC) for CH selection. Following objective functions are used to design optimal cluster formation and CH selection.

Distance is considered to be as first objective. Closeness among nodes are evaluated as in Eq. (4):

4 $$\:{Obj}_{1}=\:\mathrm{m}\mathrm{i}\mathrm{n}\left\{{\sum\:}_{i}^{1-{N}_{t}}dist\left(i,j\right)\right\}$$

Where, total nodes are represented as *N*_*t*_ and distance between i^th^ and j^th^ node as $\:dist\left(i,j\right)$. This is evaluated as Euclidean distance.

The adjacent cost function is considered as second objective which is evaluated as in Eq. (5):

5 $$\:{Obj}_{2}=\mathrm{m}\mathrm{i}\mathrm{n}\left\{\:\frac{{dist}_{mean}^{2}}{{rad}_{0}^{2}}\right\}$$

Where, $\:{dist}_{mean}^{2}$ is the mean distance among nearby nodes and radius of nodes is represented as $\:{rad}_{0}^{2}$.

The residual energy$\:{\:E}_{res}$ of nodes is one of the important objectives that need to be maximized. This is represented in Eq. (6):

6 $$\:{Obj}_{3}=\:max{\sum\:}_{i=1}^{N}{E}_{res}{(N}_{i})$$

These fitness functions are taken for evaluating the fitness function for multi-objective ensemble clustering (MOEC) algorithm.

Clustering can be thought of as the process of arranging objects in such a way that those objects that are most similar to one another are placed in the same group, while those objects that are most unlike are placed in separate groups. Formally, clustering a data collection S of N items involves finding a partition {C_1_,..., C_K_} of S such that in Eq. (7):

7 $$\:\bigcup\:_{k=1}^{K}{C}_{k}=S$$

**Fig. 4:**
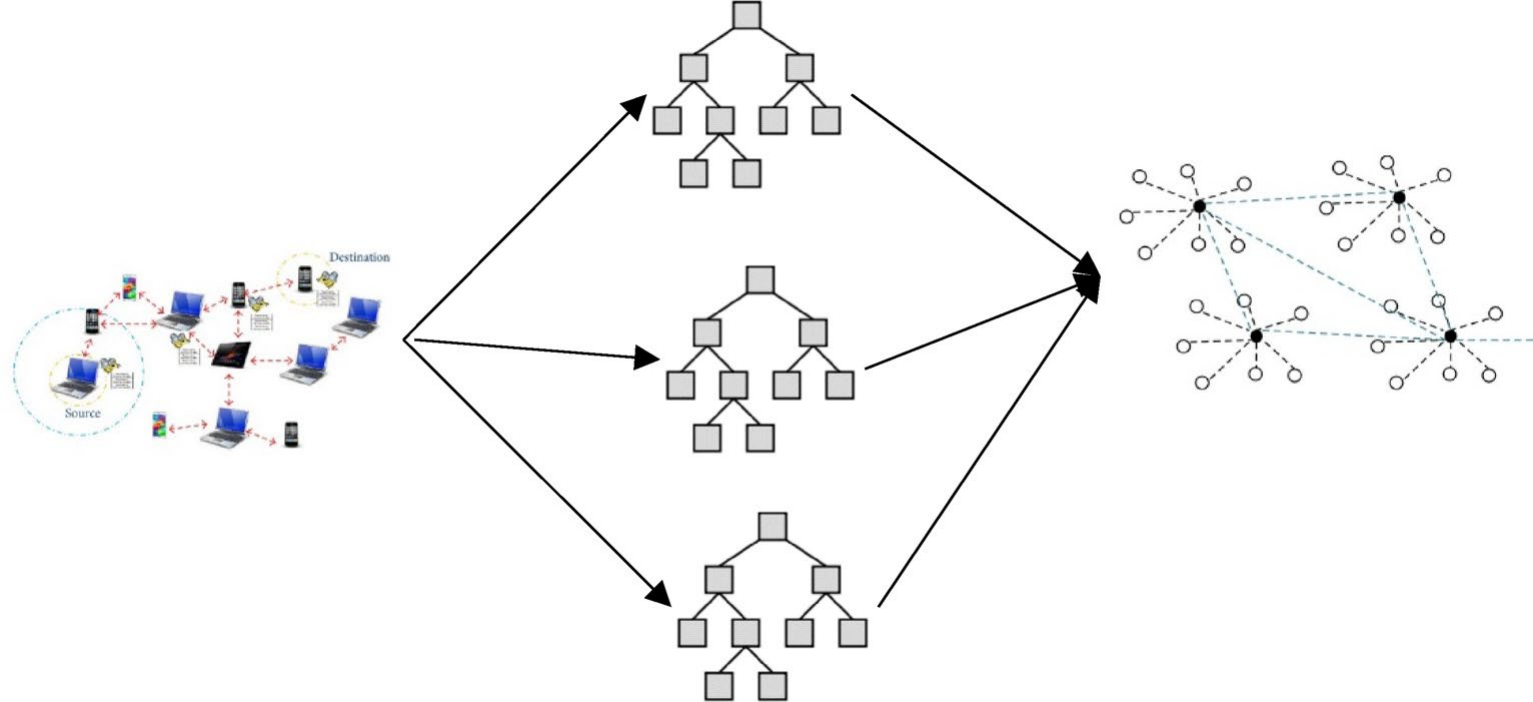
Ensemble clustering.

Most clustering techniques output partitions (disjoint clusters) as in Eq. (8):

8 $$\:{C}_{k}\cap\:{{C}_{k}}^{{\prime\:}}=\varnothing\:\:\:\:\:if\:k\ne\:{k}^{{\prime\:}}$$

which is not always desirable. Clustering is a problem that is not well defined, despite the fact that it appears to have a definite description. One of the most fundamental problems is that clustering is predicated on the assumption that items that are similar ought to be grouped together, and those that are unlike have to be split into other categories. However, in terms of mathematics, a transitive link cannot be established between similarity and belonging to the same cluster. Ensemble clustering has proved to be a popular technique in recent years, that aims to combine multiple optimization based clustering comprises of particle swarm optimization (PSO) along with Gray wolf optimization (GWO) into a better and more robust clustering. Figure 4 illustrates the core concept behind the proposed multi-objective ensemble clustering approach. Each clustering algorithm processes the input data differently. These models generate distinct clustering solutions. Instead of relying on a single algorithm’s output, the approach merges these diverse solutions into a unified consensus clustering. This consensus result is computed using ensemble techniques aimed at maximizing clustering accuracy and minimizing structural inconsistencies. The goal is to influence the strengths of different clustering algorithms and reduce the impact of their individual weaknesses. The algorithm for ensemble clustering is presented below in algorithm 1.

**Figure:**
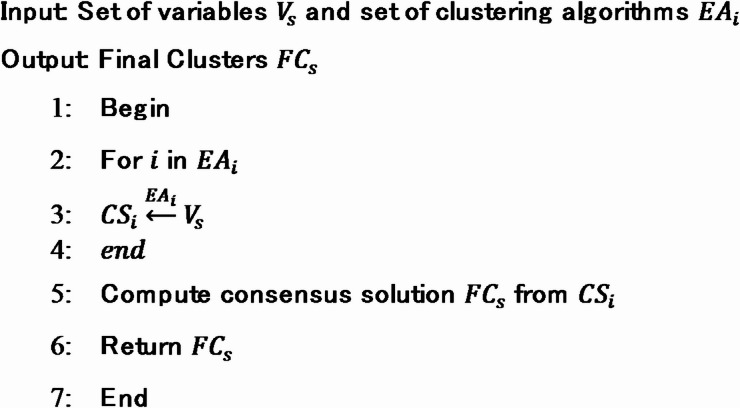
**Algorithm 1:** Multi-objective ensemble clustering

### Detection agent Initialization, attack detection and trust score generation

In highly dynamic MANETs, trust must be continuously updated to reflect fresh evidence while “forgetting” stale behavior. Practical mechanisms combine: (i) direct trust, (ii) indirect trust, and (iii) historical/aged trust with time-decay to mitigate concept drift from mobility and topology churn. A common update pattern is a Bayesian or weighted-averaging rule with aging, where recent observations are given higher weights than older ones; thresholds adapt based on link quality and congestion so that nodes are not mis-penalized for packet loss caused by poor channels rather than malice. Reinforcement-learning (RL)–assisted trust further adapts policy parameters online: nodes learn which peers to prefer for routing as rewards (PDR/throughput, energy) change with mobility and density. Trust should also be context-aware (e.g., route length, residual energy, cluster role), and safeguarded against recommendation attacks. Recent studies reinforce these design choices: Dhand et al.^42^ fuses direct, indirect and composite trust with adaptive weighting to detect malicious nodes and secure routing under mobility that demonstrates the composite trust outperforms single-source trust in dynamic environment. Similarly, Singh et al.^43^ shows that fuzzy trust + clustering lowers delay/energy while updating trust as nodes move, evidencing the value of role-aware (CH vs. member) trust aging.

In this step, each cluster is initialized with some attack detection agent nodes (ADAN) whose task is to detect the arriving traffic as normal, blackhole, wormhole and suspicious nodes. For this machine learning model are initialized. Machine learning models such as logistic regression (LR), naïve bayes (NB), XGboost (XGB), multi-layer perceptron (MLP), k-nearest neighbour (k-NN), Support vector machine (SVM).

Naive Bayes is most commonly known as a classification technique but is most probably used in the machine learning algorithm. If the output variable is discrete, then Naive Bayes should be employed. The Bayes Theorem serves as the driving force behind the algorithm’s underlying mechanisms. This is represented as in Eq. (9):

9 $$\:P\left(Y\right|X)=\frac{P\left(X|Y\right)*P\left(Y\right)}{P\left(X\right)}P\left(X|Y\right)=P\left({X}_{1}|Y\right)*P\left({X}_{2}|Y\right)*\dots\:\dots\:\dots\:*P\left({X}_{n}|Y\right)$$

Where, output variables (Y) and input variables (X).

Based on the Supervised Learning technique, K-Nearest Neighbour is the easiest ML algorithm. The main functionality of the K-NN algorithm is to assume the comparison between the new available cases and case/data and put the new instance in the group that is most analogous to the other categories that are available. Based on the similarity, the K-Nearest Neighbor Algorithm classifies a new data point and stores all the information that exists. By this study we analyze that using K- NN algorithm can be easily classified into a well suite category. The K-NN technique has many applications in Regression and it is more commonly utilized for Classification. It calculates the distance as in Eq. (10):

10 $$\:dist\:(x,z)=\left({\sum\:}_{r=1}^{d}\left|xr-zr\right|p\right)1/p$$

Where, x and z are two distinct point and p is the order of norm.

One of the most well-known Machine Learning algorithms, logistic regression is a subset of supervised learning. This technique enables the prediction of the categorical dependent variable from a set of independent variables. For a categorical dependent variable, logistic regression is used to predict the final result. The outcome must so be a discrete or categorical number. To avoid supplying the precise values of Yes and No, 1 and 0, false and true etc., it instead provides probabilistic results that range from 0 to 1. It is represented mathematically as in Eq. (11):

11 $$\:Y={b}_{0}+{b}_{1}{x}_{1\:}+{b}_{2}{x}_{2}+\dots\:\dots\:\dots\:\dots\:\dots\:{b}_{n}{x}_{n}$$

One of the most prominent instances of supervised learning is the Support Vector Machine (SVM), often known as the SVM method. Both classification and regression-related problems can be solved with the usage of this specific technique. The majority of its applications, namely Classification problems are in Machine Learning. The Support Vector Machine (SVM) technique aims at generating the optimum line or decision boundary that can categorize an n-dimensional space. As a result, it will be easy for us to categorize any new data points. A hyperplane defines optimal boundaries for making a decision as presented in Eq. (12):

12 $$\:f\left(x\right)=\sum\:_{n=1}^{N}{\alpha\:}_{n}-{\alpha\:}_{n}^{*}G\left({x}_{n},\:x\right)+b$$

Where, $\:f\left(x\right)\:$is the predicted output for input $\:x$ with N number of training samples. $\:{\alpha\:}_{n}\:and\:{\alpha\:}_{n}^{\mathrm{*}}$are Lagrange multipliers from the SVR dual optimization problem. $\:G\left({x}_{n},\:x\right)\:$is the kernel function and $\:b$ is the bias.

Multilayer Perceptrons (MLPs) are a type of feedforward artificial neural network that is fully connected. (ANN). The term “multi-layer perceptron” (MLP) is often used interchangeably with “feedforward artificial neural network” (ANN) which has been composed of multilayer perceptron. It has only one hidden layer, it is frequently referred to informally as a “vanilla” neural network. Corrections which mitigates the error in the total output for the nth data point are used to update the node weights is equated as in Eq. (13):

13 $$\:{\epsilon}_{n}=\frac{1}{2}\sum\:_{output\:node\:j}{e}_{j}^{2}\left(n\right)$$

Using gradient descent, the change in each weight w_ij_$\:A=\pi\:{r}^{2}$ is evaluated as in Eq. (14):

14 $$\:\varDelta\:{w}_{ij}\left(n\right)=-\eta\frac{\partial\:{\in\:}_{n}}{\partial\:{{v}_{j}}_{n}}{y}_{i}\left(n\right)$$

where $\:{y}_{i}\left(n\right)$ represents the output of the neuron i before it, n is the learning rate, and is chosen to ensure that the weights quickly converge to a response free of oscillations. The previous expression,$\:\frac{\partial\:{\in\:}_{n}}{\partial\:{{v}_{j}}_{n}}$, specifies the partial derivate of the error $\:{\in\:}_{n}$ in accordance with the weighted sum $\:{{v}_{j}}_{n}$ of the input connections of neuron i.

For problems with classification and regression models, extreme gradient boosting (XGBoost) is used as a class of collective machine learning techniques. Trees are simultaneously inserted into the array and matched to remedy the predicting misclassification produced by previous models. Due to its susceptibility to overfitting the XGBoost method includes normalization variables within the original GBDT algorithm. Both the accuracy as well as training time of XGBoost have been drastically enhanced in comparison to earlier methods. Let $\:{o}_{i}$ denote an output, $\:{\widehat{o}}_{i}\:$denote a predicted output, and $\:{x}_{i}\:$denote an input. Mathematically, the learning model can be represented as Eq. (15):

15 $$\:{f}_{0}\left({x}_{i}\right)={\widehat{o}}_{i}^{\left(0\right)}=0{\widehat{o}}_{i}^{\left(1\right)}={\widehat{o}}_{i}^{\left(0\right)}+{f}_{1}\left({x}_{i}\right){\widehat{o}}_{i}^{\left(2\right)}={\widehat{o}}_{i}^{\left(1\right)}+{f}_{2}\left({x}_{i}\right){\widehat{o}}_{i}^{\left(t\right)}={\widehat{o}}_{i}^{(t-1)}+{f}_{t}\left({x}_{i}\right)$$

Where, weak learning function is represented by ‘f’. The loss function, while training, is mathematically represented as in Eq. (16):

16 $$\:{loss}_{t}=\sum\:_{i=1}^{n}loss({o}_{i},{\widehat{o}}_{i})+\sum\:_{t=1}^{T}loss\left({f}_{t}\right({x}_{i}\left)\right)$$

Where, loss_t_ = training loss, $\:loss({o}_{i},{\widehat{o}}_{i})$= empirical loss between predicted and observed label, $\:loss\left({f}_{t}\right({x}_{i}\left)\right)$= loss of boosted learner.

The role of detection agent node is to predict the nature of node as malicious node (BHA or GHA), suspicious node and non-malicious node. According to prediction result nodes are assigned a trust score which is updated regularly. The range of trust value is considered as [0–4], where initial trust is assigned to be as 2 to all nodes. If any node is determined by agent as normal then its trust score $\:{S}_{trust}$ is increased and its score is $\:>2$ and if any node is determined as suspected then it’s trust value is decreased by 1. If any node is predicted as malicious node, then its trust value is set to 0. For evaluation of $\:{S}_{trust}$, The a determining factor $\:{Q}_{trust}\:$is evaluated after every direct and indirect communication and calculated as in Eq. (17):

17 $$\:{Q}_{trust}=\frac{Total\:successful\:transaction\:by\:ith\:nodes}{Total\:transaction\:by\:ith\:node}$$

Where, if any node whose $\:{Q}_{trust}$ is greater than 0.5 then it is not required to predict its nature whereas if any node whose $\:{Q}_{trust}$ is less than 0.5 then that node is under vigilance by agent node ADAN. This will reduce the computational complexity of the node. The algorithm of attack detection and trust score generation is presented in algorithm 2.

**Figure:**
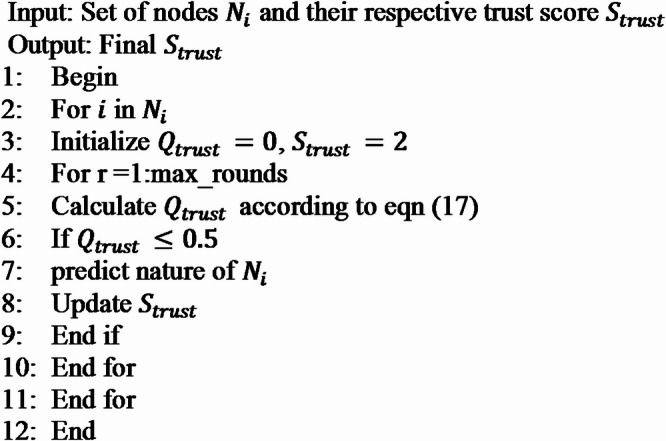
**Algorithm 2:** Attack detection and trust score generation.

### Attack prevention

In this stage, nodes that are identified as malicious node are added to malicious node list. During data transmission, these are nodes are removed from route discovery phase of data transmission.

## Results and discussions

In this section, result and evaluation is presented for proposed model. This section has been segregated into sub-Sect. 5.1 describes the dataset used for the designed model. Then Sect. 5.2 describes the result evaluation of machine learning approaches for attack detection. Then Sect. 5.3 presents the result of proposed model under attack condition. Finally, in Sect. 5.4 the paper presents the comparative state-of-art.

### Dataset description

**Fig. 5:**
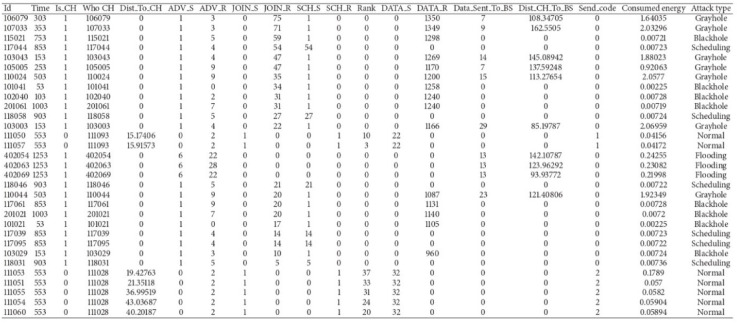
Data sample from WSN-DS dataset^35^.

**Fig. 6:**
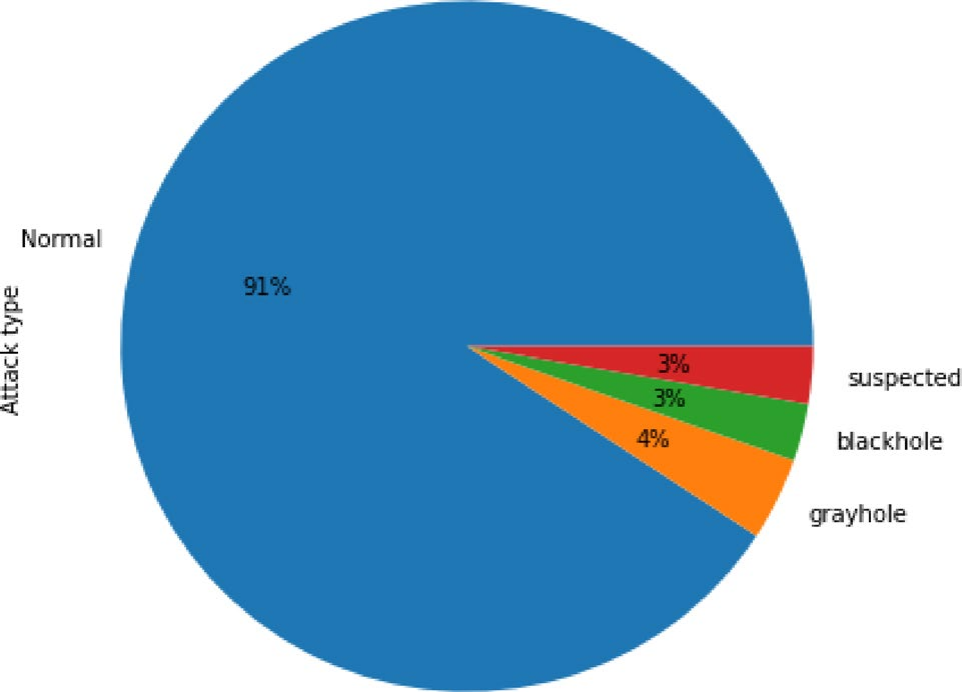
Distribution of attacks in WSN-DS dataset.

In this work, we have implemented WSN-DS dataset for training of model which was collected from [35, https://www.kaggle.com/datasets/bassamkasasbeh1/wsnds] whose samples are presented in Fig. 5. The dataset for training is converted into four categories i.e., normal, blackhole, gray hole and suspected. The distribution of data samples is presented in Fig. 6. The chart shows that the overwhelming majority of the data, 91%, represents normal (benign) network behaviour, which reflects the real-world condition where malicious activity occurs less frequently but has significant impact. Among the attack classes, gray hole attacks account for 4%, where malicious nodes selectively drop packets. Both blackhole attacks and suspected nodes each contribute 3% to the dataset.

### Performance of ML for BHA or GHA attack detection

In this section, result analysis of the attack detection model is presented for which accuracy, precision, recall and f1_score has been utilised for evaluating the performance. Mathematically, they are represented as in Eq. (18)-Eq. (21):

18 $$\:Accuracy\:=\:\frac{TruePositive+TrueNegative}{TruePositive+TrueNegative+FalsePositive+FalseNegative}$$

19 $$\:Precision=\frac{TruePositive}{TruePositive+FalsePositive}$$

20 $$\:Recall=\frac{TruePositive}{TruePositive+FalseNegative}$$

21 $$\:F1\_score=\frac{2}{1/precision+1/Recall}$$

The results presented in Table 2 clearly indicate that XGBoost (XGB) outperforms all other models across all metrics. This demonstrates its superior ability to correctly classify both normal and attack behaviors in the network. However, algorithms such as Naïve Bayes, Logistic Regression, and MLP showed relatively lower performance indicating higher rates of false positives or less reliable classification under certain conditions.

**Table 2:** Performance of different machine learning algorithms for attack detection and Prevention.

Algorithm	Accuracy	Precision	Recall	F1-Score
NB	0.87	0.84	0.87	0.86
XGB	0.99	0.99	0.99	0.99
k-NN	0.96	0.95	0.96	0.96
LR	0.91	0.83	0.91	0.86
SVM	0.96	0.92	0.96	0.94
MLP	0.91	0.82	0.91	0.86

**Fig. 7:**
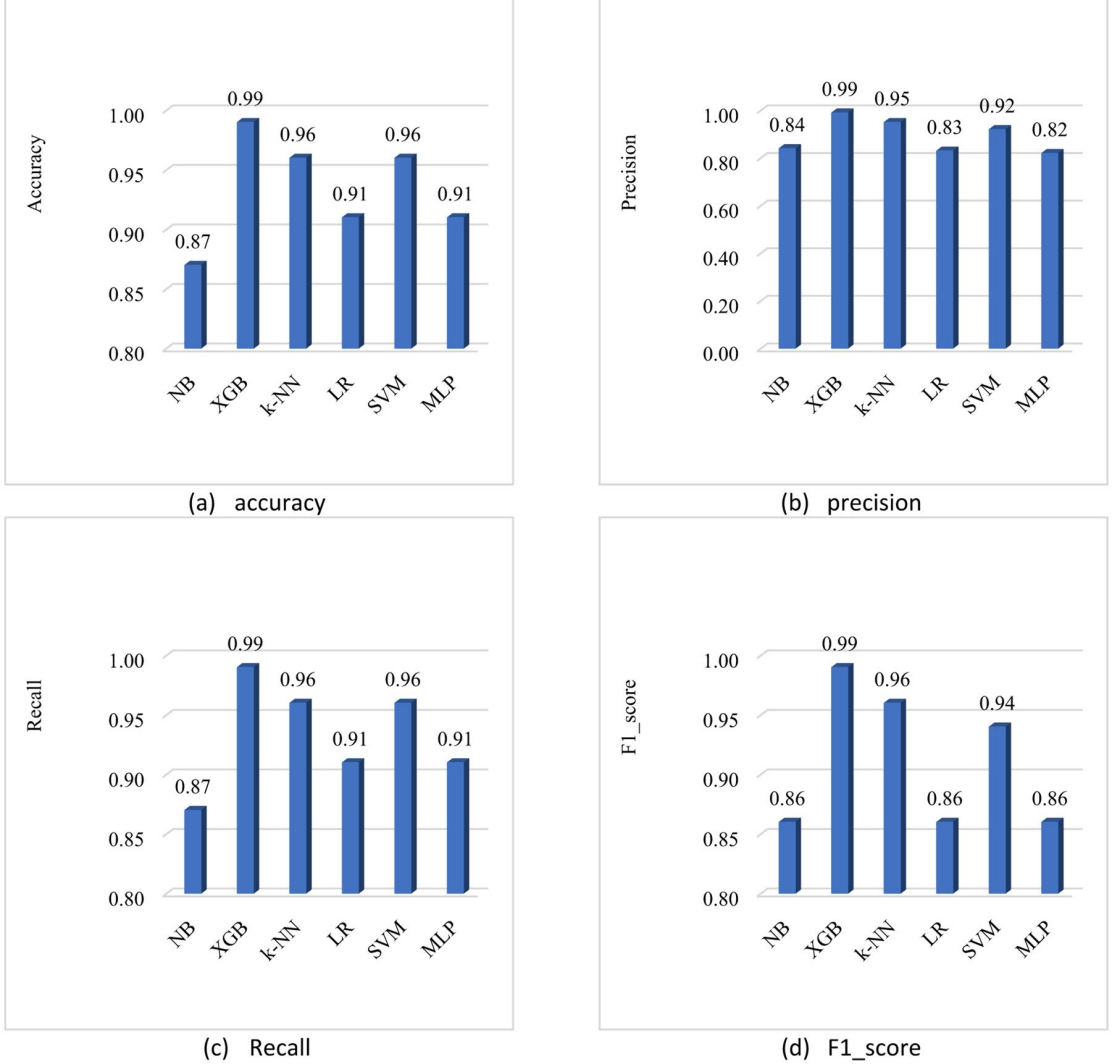
Performance comparison of ML approaches for attack detection.

Different machine learning models and their corresponding accuracy scores is shown in 7(a) to 7(d). The Fig. 7(a) shows the accuracy scores for different machine learning models that are used for attack detection. NB (Naive Bayes) has an accuracy of 0.87, XGB (Extreme Gradient Boosting) has an accuracy of 0.99, k-NN (k-Nearest Neighbors) has an accuracy of 0.96, LR (Logistic Regression) has an accuracy of 0.91, SVM (Support Vector Machine) has an accuracy of 0.96, MLP (Multi-Layer Perceptron) has an accuracy of 0.91. The Fig. 7(b) shows the precision scores for different machine learning models that are used for attack detection. The precision scores for the different models. NB has a precision of 0.84 XGB has a precision of 0.99, k-NN has a precision of 0.95, LR has a precision of 0.83, SVM has a precision of 0.92, MLP has a precision of 0.82. Based on precision alone, XGB seems to be the best-performing model, followed by k-NN and SVM. The Fig. 7(c) shows the recall scores for different machine learning models that are used for attack detection. NB has a recall of 0.87, XGB has a recall of 0.99, k-NN has a recall of 0.96, LR has a recall of 0.91, SVM has a recall of 0.96, MLP has a recall of 0.91. Based on recall alone, XGB seems to be the best-performing model, followed by k-NN and SVM in terms of recall. Figure 7(d) shows the F1-score for different machine learning models. NB has an F1-score of 0.86, XGB has an F1-score of 0.99, k-NN has an F1 score of 0.96, LR has an F1-score of 0.86, SVM has an F1-score of 0.94 and MLP has an F1-score of 0.86. Based on the F1-score alone, XGB seems to be the best-performing model, followed by k-NN and SVM.

**Fig. 8:**
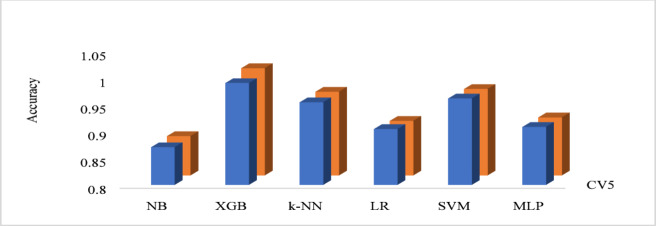
Cross-validation result for ml approaches for attack detection.

**Fig. 9:**
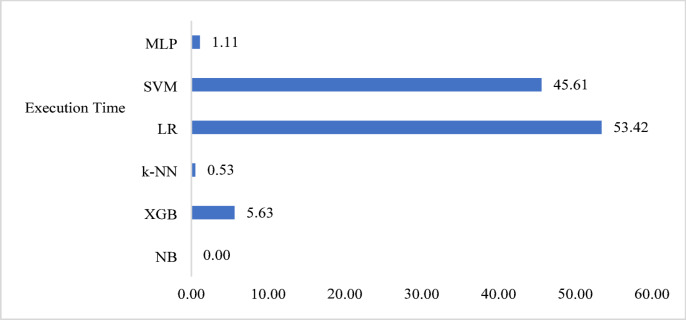
Execution time comparison of ml approaches for attack detection.

Figure 8 shows the cross-validation (CV) scores for different machine learning models using two different cross-validation techniques: 5-fold cross-validation (CV5) as well as 10-fold cross-validation (CV10). The performance of machine learning models is frequently evaluated and compared taking the usage of the cross-validation technique. The data is divided multiple times during cross-validation, and various subsets of the data are implemented to train and test the model. NB has a CV5 score of 0.87 and a CV10 score of 0.873. XGB has a CV5 score of 0.99 and a CV10 score of 1. k-NN has a CV5 score of 0.954 and a CV10 score of 0.956. LR has a CV5 score of 0.904 and a CV10 score of 0.9015. SVM has a CV5 score of 0.9612 and a CV10 score of 0.9612. MLP has a CV5 score of 0.908 and a CV10 score of 0.908. Based on the scores, it appears that XGB and SVM have the highest cross-validation scores across both CV techniques. Figure 9 shows the execution time (in seconds) comparison of different machine learning (ML) models for attack detection. Execution time is an important consideration when selecting a ML approach as some models may be computationally expensive, requiring more time to train and predict than others. Based on the execution time alone, XGB seems to be the optimal performing model.

### Result analysis

In this section, the result evaluation of DoS attack detection along with prevention in MANET. The entire network is simulated on MATLAB. The simulation setup is demonstrated in Table 3. For simulation random location of sensor nodes are considered and are deployed with limited energy.

**Table 3:** Simulation configuration.

Parameters	Values
Area	100 m*100m
No. of Sensor nodes	Variable
Initial Energy of network	5 J
Energy Dissipation while receiving bits	50 nJ/bits
Energy Dissipation while transmitting bits	50 nJ/bits
Packet size	Variable

**Fig. 10:**
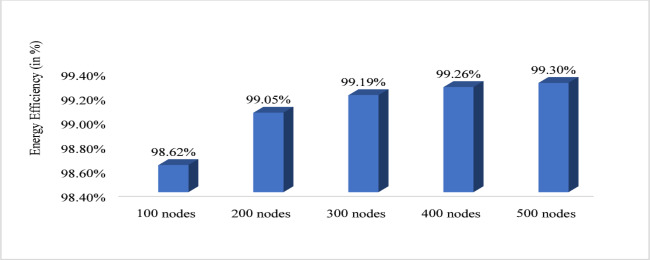
Energy efficiency with respect to node density.

**Fig. 11:**
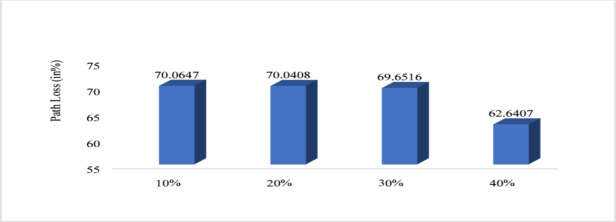
Path loss respect to attack ratio.

**Fig. 12:**
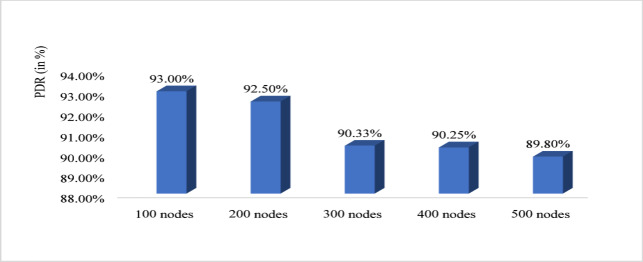
Packet delivery ratio with respect to node density under attack.

**Fig. 13:**
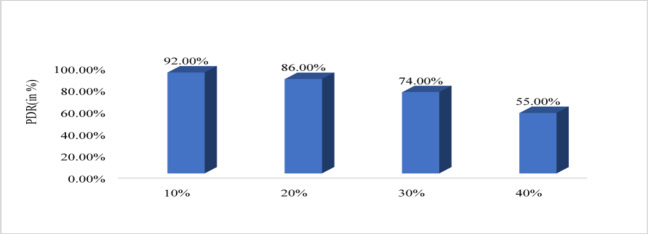
Packet delivery ratio with respect to attack ratio.

**Fig. 14:**
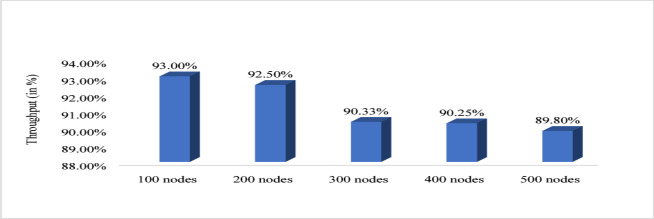
Throughput with respect to node density under attack.

**Fig. 15:**
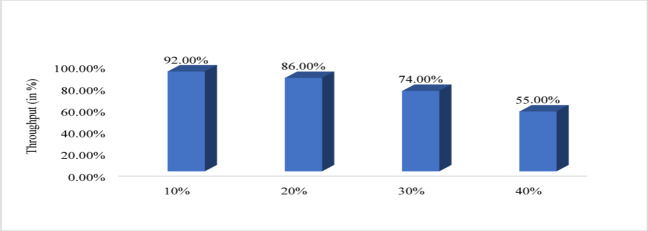
Throughput with respect to attack ratio.

Figure 10 shows that energy efficiency with respect to node density. The nodes vary from 100 to 500 and energy efficiency is evaluated. For 100 nodes energy efficiency is minimum that is 98.62%, for 200 it is 99.05%, for 300 it is 99.19%, for 400 it is 99.2 6% and for 500 it is maximum that is 99.30%. Therefore, from graph it is observed that with increased node the proposed system increases the energy efficiency. Figure 11 shows path loss respect to attack ratio that varied from 10 to 40% of total node. The path loss for 10% attack ratio is 70.06, for 20% attack ratio it is 70.04%, for 30% ratio it is 69.65 and for 40% attack ratio it is 62.64% which is minimum. Figure 12 shows the packet delivery ratio with respect to node density under attack. For 100 node PDR is 93%, for 200 PDR is 92.5%, for 300 PDR is 90.3%, for 400 PDR is 90.25% and for 500 PDR is 89.80%. It is clearly visible that as the nodes increasing the PDR ratio decreases and therefore with increasing nodes, the PDR is decreasing. Figure 13 shows packet delivery ratio with respect to attack ratio. The PDR for 10% at attack ratio is 92%, for 20% attack ratio PDR efficiency is 86%, for 30% attack ratio PDR efficiency is 74% and for 40% attack ratio PDR efficiency is 55%. It is clear from the graph that as the attack ratio rises the PDR decreases, we can say that PDR and attack ratio as inversely proportional. Figure 14 shows the throughput with respect to node density under attack ratio. For 100 nodes throughput is 90%, for 200 nodes throughput is 92.50%, for 300 nodes it is 90.33%, for 400 nodes it is 90.25% and for 500 nodes it is 89.80% which is minimum in all. Figure 15 shows the throughput with respect to attack ratio where is from 10% to 40%. For 10% it is 92%, for 20% it is 86%, for 30% it is 74% and for 40% it is 55%. Clearly shown from the pattern of the graph that, as the attack ratio increases the throughput decreases.

Table 4 presents the comparative analysis of the proposed technique comprehensively that outperforms existing methods^36^-^40^ by incorporating features presented in table. While previous methods consistently address Blackhole Attack Detection (BHAD) and Detection Systems (DS) but they lack uniform support for features like EEC, TM, PS and scalability. Thus, the proposed method uniquely fills critical gaps by providing an integrated, secure, energy-efficient, and scalable solution.

**Table 4:** Comparative feature Analysis.

Techniques	EEC	BHAD	GHAD	TM	ML	DS	PS	S
^36^	×	√	√	×	√	√	√	×
^37^	×	√	√	×	√	√	×	√
^38^	×	√	√	×	√	√	×	×
^39^	×	√	×	×	×	√	√	√
^40^	×	√	×	×	×	√	√	×
Ours	√	√	√	√	√	√	√	√

*EEC = Energy efficient clustering*,* BHAD = Blackhole attack detection*,* GHAD = Gray hole attack detection*,* TM = Trust management*,* DS = Detection System*,* PS = Prevention System*,* Scalable.*

Below in Table 5, average throughput and average delay is comparative state of art is presented.

**Table 5:** Comparative State-of-Art.

Techniques	Average throughput (Kbps)	Average delay
Rani et al^37^.	85.64	0.150
Zardari et al^38^.	-	0.059
Dani et al^40^.	45	0.010
Ours	93	0.003

**Fig. 16:**
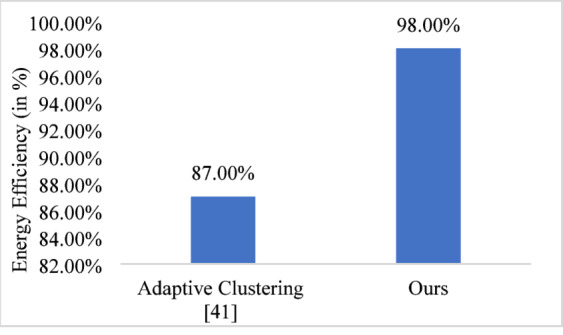
Comparative energy efficiency.

The proposed approach demonstrates superior energy efficiency compared to existing adaptive clustering^41^ as presented in Fig. 16 by achieving an energy efficiency of approx. 98% and approx. 87% respectively for 100 nodes. This reflects a 11% improvement in energy utilization. This enhancement is attributed to optimized power allocation, reduced control overhead, and intelligent clustering strategies that enable more effective energy distribution across nodes. This ensures prolonged network lifetime and better resource management, making the proposed solution more energy-efficient and sustainable for large-scale deployments.

## Conclusion

In this paper, an attack detection and prevention model are presented. The paper demonstrated the model on DoS attacks including Gray hole and Blackhole attacks. The paper designed covered three issues related with MANET, energy-efficiency, attack detection and prevention. To enhance energy efficiency, the system employs an ensemble-based clustering optimization approach for optimal cluster head selection, minimizing energy consumption during data aggregation. For security, the model integrates agent-based attack detection, where dedicated nodes within each cluster monitor network traffic in real-time. Among the models tested, XGB demonstrated the highest detection performance. To mitigate the impact of malicious nodes post-detection, the system assigns trust scores, which guide routing and prevent future exploitations. Following points are concluded from this research work:

- A hybrid framework combining clustering, agent-based monitoring, and machine learning that is capable to detect DoS attacks in MANET.
- The proposed system is energy efficient and reduces the packet drop ratio as compared to others. This shows improvement in network lifetime and reducing overhead.
- A trust-based mitigation mechanism, enhancing the resilience of MANETs post-detection.
- The proposed system can detect and prevent the attack-prone MANET environment.

The results indicate that the proposed approach significantly reduces packet drop rate, improves throughput, and lowers average delay, outperforming existing state-of-the-art solutions. However, when developing such systems, it is important to take into account the drop in packet delivery ratio with more nodes. This can be extended as future scope. Although the system performs well, future research should address scalability issues, optimize models for edge deployment, expand attack coverage, and explore decentralized trust mechanisms to further enhance MANET flexibility.

## Data Availability

All data generated or analyzed during this study are included in this published article.
